# Semi-automated software improves interrater reliability and reduces processing time of magnetic resonance imaging-based exocrine pancreatic assessments in pediatric patients

**DOI:** 10.1007/s00261-024-04442-1

**Published:** 2024-06-19

**Authors:** Jonathan A. Dudley, Nadeen Abu Ata, Kyle E. Murdock, David S. Vitale, Maisam Abu-El-Haija, Andrew T. Trout

**Affiliations:** 1 Imaging Research Center, Cincinnati Children’s Hospital Medical Center, 3333 Burnet Avenue, Cincinnati, OH 45229-3026 USA; 2 Department of Radiology, Cincinnati Children’s Hospital Medical Center, Cincinnati, OH USA; 3 Division of Gastroenterology Hepatology and Nutrition, Cincinnati Children’s Hospital Medical Center, Cincinnati, OH USA; 4 Department of Pediatrics, College of Medicine, University of Cincinnati, Cincinnati, OH USA; 5 Department of Radiology, University of Cincinnati College of Medicine, Cincinnati, OH USA

**Keywords:** Pancreatic function tests, Cholangiopancreatography, Magnetic resonance imaging, Observer variation

## Abstract

**Objectives:**

Magnetic resonance (MR) imaging with secretin stimulation (MR-PFTs) is a non-invasive test for pancreatic exocrine function based on assessing the volume of secreted bowel fluid in vivo. Adoption of this methodology in clinical care and research is largely limited to qualitative assessment of secretion as current methods for secretory response quantification require manual thresholding and segmentation of MR images, which can be time-consuming and prone to interrater variability. We describe novel software (PFTquant) that preprocesses and thresholds MR images, performs heuristic detection of non-bowel fluid objects, and provides the user with intuitive semi-automated tools to segment and quantify bowel fluid in a fast and robust manner. We evaluate the performance of this software on a retrospective set of clinical MRIs.

**Methods:**

Twenty MRIs performed in children (< 18 years) were processed independently by two observers using a manual technique and using PFTquant. Interrater agreement in measured secreted fluid volume was compared using intraclass correlation coefficients, Bland-Altman difference analysis, and Dice similarity coefficients.

**Results:**

Interrater reliability of measured bowel fluid secretion using PFTquant was 0.90 (0.76–0.96 95% C.I.) with − 4.5 mL mean difference (-39.4–30.4 mL 95% limits of agreement) compared to 0.69 (0.36–0.86 95% C.I.) with − 0.9 mL mean difference (-77.3–75.5 mL 95% limits of agreement) for manual processing. Dice similarity coefficients were better using PFTquant (0.88 +/- 0.06) compared to manual processing (0.85 +/- 0.10) but not significantly (*p* = 0.11). Time to process was significantly (*p* < 0.001) faster using PFTquant (412 +/- 177 s) compared to manual processing (645 +/- 305 s).

**Conclusion:**

Novel software provides fast, reliable quantification of secreted fluid volume in children undergoing MR-PFTs. Use of the novel software could facilitate wider adoption of quantitative MR-PFTs in clinical care and research.

## Introduction

Exocrine pancreatic function, wherein bicarbonate and digestive enzymes are secreted from the pancreas in response to food within the duodenum, is required for digestion and absorption of food particles and certain micronutrients including fat-soluble vitamins. Exocrine pancreatic insufficiency (the inability for the pancreas to make enough of the digestive enzymes) is associated with weight loss and steatorrhea in children and adults and poor growth and development in children [1]. Reference standard testing for exocrine function requires either collection of stool for quantification of fecal elastase (indirect test) or endoscopy with enteric fluid collection (endoscopic pancreatic function testing [ePFT]) to measure bicarbonate concentration and/or enzyme function (direct test) [2–4].

MR-pancreatic function testing (MR-PFT) [5], wherein T2-weighted images are acquired subsequent to administration of a secretagogue and the volume of fluid secreted is subjectively graded or quantitatively measured, have been described as a non-invasive method of exocrine pancreatic function testing. The most common method of assessment of pancreatic function based on MR-PFT relies on the Matos criteria, with qualitative assessment of the degree of duodenal filling [5]. Matos grade has been linked to pancreatic exocrine function in adults [6]. Although assessment of pancreatic function via the Matos criteria is clinically utilized in day-to-day practice, the assessment is subjective and may not be appropriate for pediatric patients [7]. Instead, the quantitation of secreted fluid volume in response to administration of a secretagogue may represent a more accurate and generalizable test for exocrine pancreatic function in both children and adults [6, 8–10]. Threshold values for normal secreted fluid volume have been defined for both adults and children [8, 11], but this quantitative approach has yet to enjoy widespread adoption. This, in part, reflects the manual effort and time required to segment images pre- and post-secretagogue and likely also reflects lack of validation of diagnostic thresholds for exocrine insufficiency, particularly for children. Validation of previously defined diagnostic thresholds necessitates tools to rapidly and reliably quantify secretory function measured by MR-PFTs.

Current methods of quantifying secretory response in MR-PFTs rely on image thresholding or other means of segmenting fluid pixels within MRI images [8, 10], a largely manual, time-consuming task. Manual image segmentation is also associated with interrater variability [10]. This variability encompasses differences in image windowing and leveling, in the threshold applied, and in region of interest placement, among other factors. With two experienced radiologists segmenting images after careful co-training, the average difference in measured fluid volume was approximately 2 mL but with 95% limits of agreement of +/- 40 mL [10]. In real-world clinical practice, clinically employed image segmentation tasks are often performed by clinical image analysts in so-called “3D labs.” Variability in measured fluid volume in this environment is expected to be greater.

To increase the utility of MR-PFTs and to enable application, we set out to develop software to facilitate fluid volume quantification by reducing the time required to perform this task and reducing interrater variability in measured fluid volume. Our software solution aimed to accomplish this by (1) automating initial thresholding of the images, (2) automatically detecting and removing hyperintense voxels that are not bowel fluid, and (3) providing semi-automated interactive tools for refinement that encourage consistent, data-driven contours. Herein, we detail the processing steps for (A) current standard methods of quantifying secretory response and (B) our proposed software solution. We also compare the performance of the two methods using existing clinical MRI examinations obtained with MR-PFT.

## Methods

Institutional review board approval was received for this retrospective study with a waiver of documentation of informed consent.

### MR exams

We searched our clinical picture archiving and communication system (PACS) (Merge PACS; Merative; Ann Arbor, MI) for clinically-obtained MRI examinations for use in this work. Inclusion criteria were: (1) Examination performed between January 15 and June 15, 2023; (2) Examination performed on a Philips MRI machine; (3) Examination performed with secretin administration; (4) Patient age < 18 years at the time of imaging. This query returned a set of 38 examinations (11 at 3T, 27 at 1.5T), from which 10 examinations at each MRI field strength were randomly selected to be used in the comparative analyses herein. MRI examinations were routed from the clinical PACS server to a secure network storage location accessible to the study team.

All MR examinations had been acquired on Philips Ingenia scanners. The acquired MR-PFT series used a T2-weighted single shot spin echo sequence with respiratory triggering. Acquisition parameters were precisely matched for the pre- and 15-minute post-secretin images and were as follows for the analyzed examinations: TE = 140ms, flip angle = 90°, slice thickness = 4 mm. Field-of-view ranged from 220 × 220 mm to 350 × 350 mm depending on patient size, acquisition matrices ranged from 256 × 256 to 400 × 400, and in-plane resolution ranged from 0.78 × 0.78 mm to 0.88 × 0.88 mm.

### Standard image processing

For each pair of MR-PFT series, bowel fluid volume pre- and post-secretin administration was quantified via manual segmentation using ImageJ software (https://imagej.net/software/imagej) by a board-certified pediatric radiologists (ATT, 11-years experience) and a PhD advanced image analyst (JAD, 11-years experience). The raters will be referred to as R1_standard_ and R2_standard_ from hereon. Each rater recorded their time to complete each exam using a stopwatch app on their phone; segmentation was performed in accordance with the following instructions:

1. Import pre- and post-secretin DICOM series.
2. Manually adjust window level for a single image and apply to all images.
3. Combine image stacks.
4. Reduce image intensity to 8-bit depth.
5. Duplicate stack.
6. Threshold image; manual selection guided by subjective assessment that all fluid voxels are thresholded.
7. Remove areas that are not bowel using the duplicated stack for anatomical reference.
8. Save the final segmented combined stack as a (lossless) multi-layer tif file.

After all examinations had been processed, the segmented image stacks were loaded into MATLAB (MathWorks; Natick, MA) as binary arrays for quantification of fluid volumes. Pre- and post-secretin volumes were calculated as the sum of pixels in the left and right half of the image stacks, respectively, multiplied by the product of the slice thickness and pixel spacing values obtained from the DICOM metadata tags.

### Semi-automated processing (PFTquant software)

For each examination, bowel fluid volume pre- and post-secretin administration was quantified via semi-automated manual segmentation by the same two raters described above. The raters will be referred to as R1_PFTquant_ and R2_PFTquant_ from hereon. To minimize potential sources of systematic bias, the following conditions were set: (1) R1 performed manual segmentations prior to semi-automated segmentation whereas R2 performed semi-automated segmentations prior to manual segmentations. (2) Neither rater could view any segmentation results prior to completing all segmentations using both approaches. (3) For each examination, an interval of at least 4 weeks separated the processing approaches of each rater in order to minimize recall of specific segmentation choices for a given examination. (4) Three different MR exams that were not among the 20 selected for this study were used in the development of the software.

**Fig. 1:**
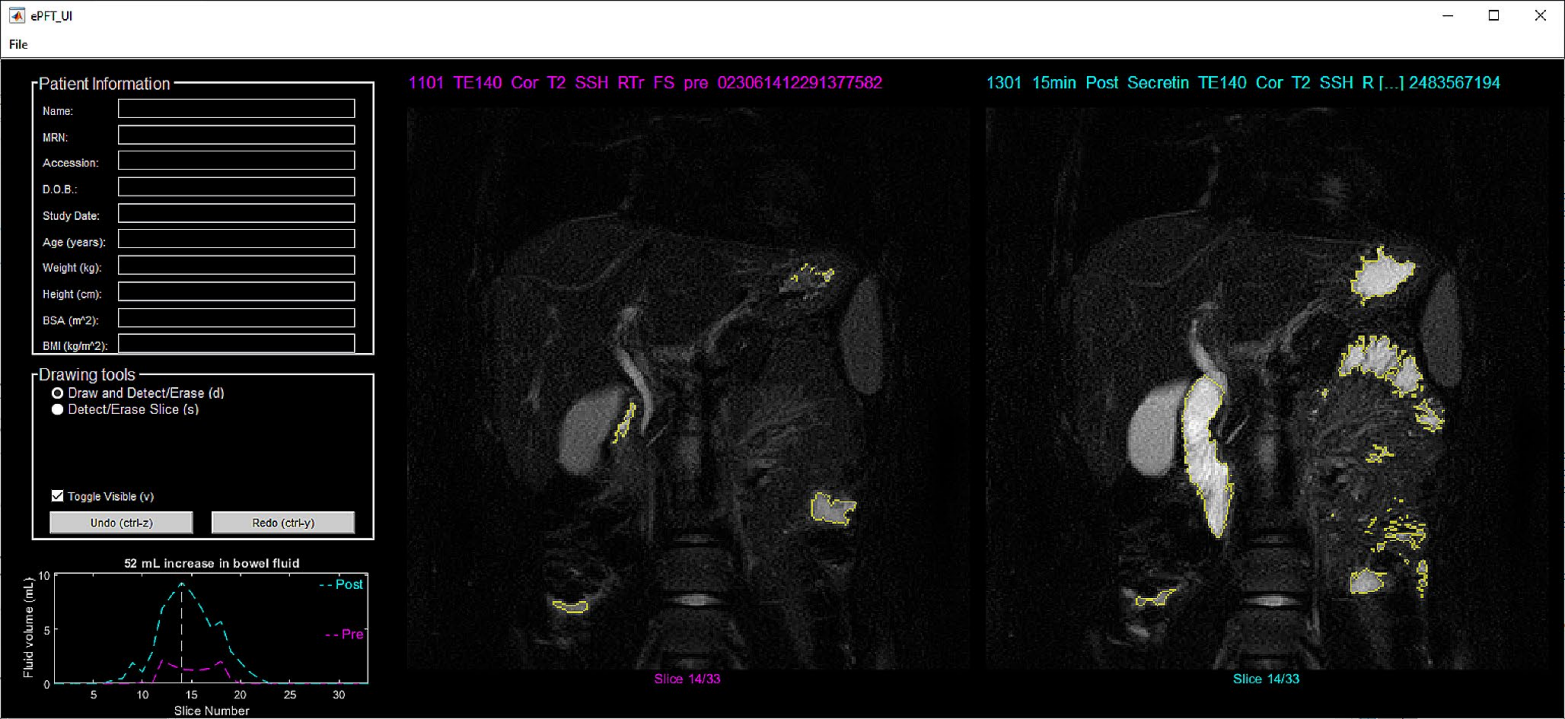
PFTquant software interface. Coronal fat-saturated images from the T2-weighted scans prior to (left image) and 15 min following (right image) secretin administration with segmented bowel fluid outlined in yellow. The upper left panel is for the display of patient information which has been redacted here. The middle-left panel contains tools for the user to edit segmentations. The lower left panel plots the pre- and post-secretin bowel fluid volumes of each slice as a fluid volume curve with the volume difference reflecting the secretory response

The user interface of the software is shown in Fig. 1. Time to complete processing of each examination was recorded by the software; the timer started the moment the user initiated a new case and stopped when the case was saved and closed (no case was re-opened for editing).

Upon selection of the pre- and post-secretin series, the software processes the data in the following manner: First, images and their corresponding metadata are loaded into memory from their DICOM files and then pre- and post-images are converted to single-precision grayscale images with the top 0.001% of voxel intensity values clipped to a value of 1. Next, pre- and post-secretin bowel-fluid candidate voxels are selected and stored in 3D binary arrays by thresholding at the intensity level that maximizes the inter-class entropy across all slices of both pre- and post-images. From this set of candidate voxels, clusters of volume less than 1 mL are removed. Finally, several non-bowel fluid objects are identified and removed based on the morphometric properties of the clusters to which they belong. This step is accomplished by resampling candidate voxel arrays to isotropic spacing before calculating morphometric properties of each cluster (e.g., centroid, bounding boxes, sphericity, angle of the principal axis of ellipsoid) and comparing those properties to sets of heuristically determined ranges for each of several non-bowel fluid objects. Non-bowel fluid objects targeted for identification are spinal canal, intervertebral discs, renal pelvis and proximal ureter, bladder, gallbladder, and incompletely saturated fat signal.

Remaining candidate voxels are mapped onto the pre- and post-secretin images of the PFTquant interface (demarcated by marginal lines tracing their boundary) for manual refinement by the rater as shown in Fig. 1. The PFTquant allows the rater to scroll through slices using the mouse wheel or left and right arrow keys. The rater may add voxels to the bowel fluid segmentation in two ways. (1) Single click – the rater simply uses the *left* mouse button to click on a point in the image within the bounds of an area of fluid. This initiates the Chan-Vese automated active-contouring algorithm which detects object boundaries by minimization of an energy function using the selected point as a starting seed [12]. (2) Regional selection – the rater presses and holds the *left* mouse button to draw a freeform shape encompassing the fluid and any amount of background. This performs a maximum entropy threshold operation limited to the voxels contained within the freeform shape. Similarly, the rater may remove voxels from the bowel fluid segmentation in two ways: (1) Single click – the rater simply uses the *right* mouse button to click on a point in the image within the bounds of an area of fluid. This initiates the aforementioned automated active-contouring algorithm using the selected point as a seed and removes the identified voxels from the segmentation. (2) Regional selection – the rater presses and holds the *right* mouse button to draw a freeform shape. All voxels within the shape are removed from the segmentation. Pre- and post-secretin volumes are calculated as the sum of the segmented voxels of their respective image multiplied by the product of the slice thickness and pixel spacing values obtained from the DICOM metadata tags. Volumes are updated actively based on user modification of the fluid segmentation. Once the rater has completed the segmentation, they may save an analysis file that contains the raw image data, the image metadata, segmented bowel fluid masks, measured pre- and post-secretin bowel fluid volume, and analysis metadata (e.g., time to process, software version).

### Statistical analyses

Interrater reliability was assessed for the outcome measure of pre- to post-secretin change in bowel fluid volume (in mL) using the two-way random effects intraclass correlation coefficient for absolute agreement between single raters, also known by the convention ICC(2,1) [13]. Results were interpreted as: ICC < 0.5 indicating poor agreement, ICC = 0.5–0.75 indicating moderate agreement, ICC = 0.75–0.9 indicating good agreement, and ICC > 0.90 indicating excellent agreement [13]. Bland-Altman difference analyses were also performed to quantify the difference in measured fluid volume between observers.

While change in bowel fluid volume is the clinical metric of interest, it is conceivable that two raters could produce the same result for an exam despite identifying vastly different sets of voxels as bowel fluid. Accordingly, we also computed the Dice similarity coefficient (DSC) as an alternative metric of interrater agreement. DSC is a measure of spatial overlap ranging from 0 (indicating no overlap in segmentation results) to 1 (indicating perfect overlap in segmentation results. Statistical inferencing was performed using a paired samples t-test on the logit transformed DSC. The logit transform, defined as logit(DSC) = ln[DSC/(1-DSC)] maps the range of [0,1] to a normal distribution with range (-∞,∞).

Agreement analyses were performed for the following analysis pairs: R1_standard_:R2_standard_, R1_PFTquant_:R2_PFTquant_ to characterize agreement between observers performing manual processing and observers performing semi-automated processing respectively.

Finally, we tested whether the PFTquant software enabled more efficient processing of the images by comparing the mean time to process images for each method via an independent t-test with *p* < 0.05 considered statistically significant. All statistical tests were performed using MATLAB R2022b (MathWorks, Natick, MA).

## Results

A total of 20 exams from 20 unique patients were included. Patient median age was 11.2 years (IQR: 8.7–15.2). The youngest patient was 2.7 years old. Twelve (60%) of the 20 patients were female. All patients underwent MRCP with secretin for clinical indications and 18 out of 20 were diagnosed with some form of pancreatic disease: 7 acute pancreatitis, 2 acute recurrent pancreatitis, 6 chronic pancreatitis, 2 exocrine pancreatic insufficiency, 1 fatty pancreas, 1 Caroli’s disease, and 1 choledochal cyst.

**Fig. 2:**
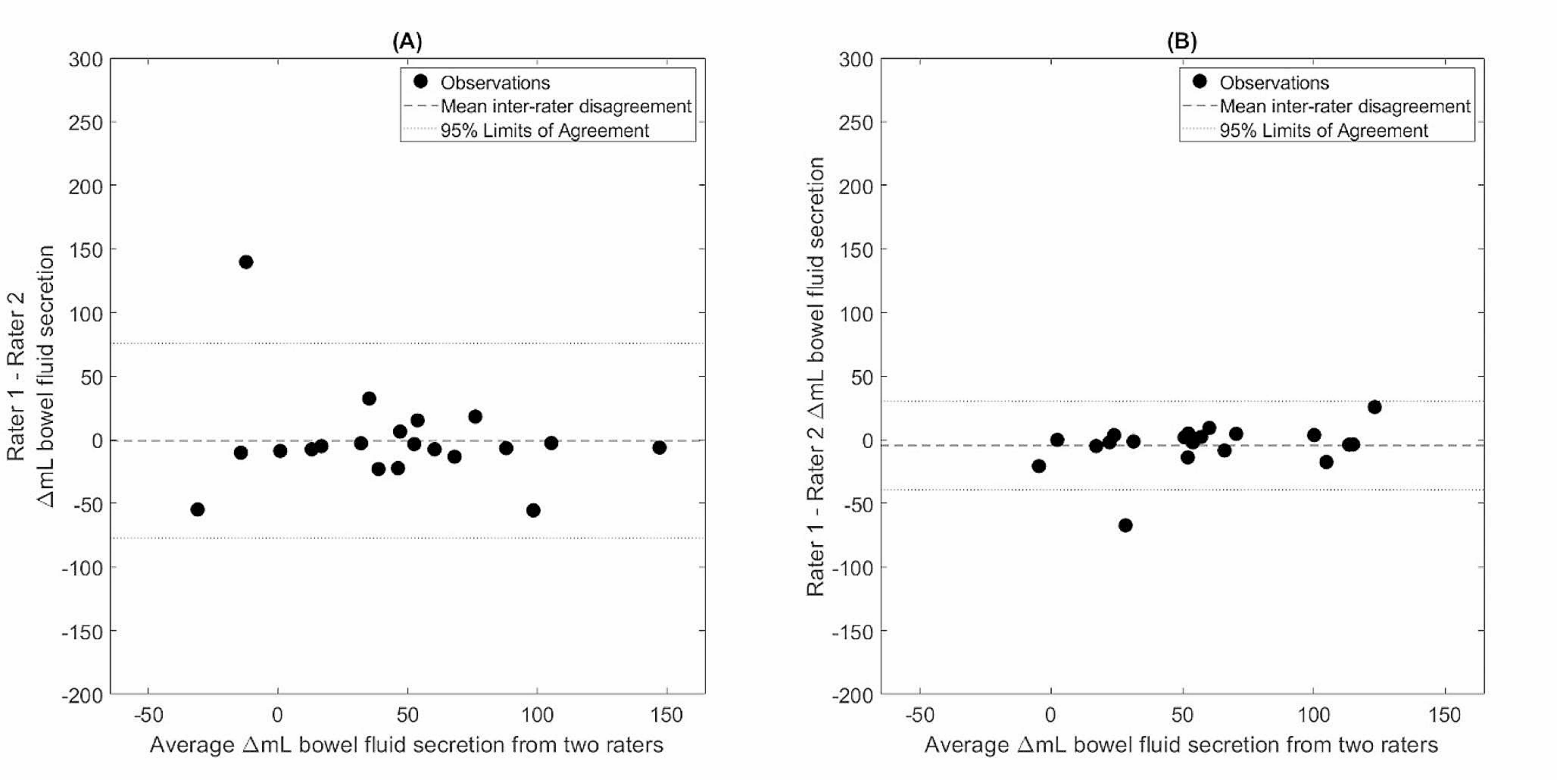
Bland-Altman difference plots for the manual (**A**) and semi-automated PFTquant (**B**) methods of quantifying bowel fluid secretion. Plots are shown with the same scale. The dashed line indicates the mean difference between raters for secreted fluid volume, calculated as post- minus pre-secretin bowel fluid volumes. The dotted lines indicate the 95% limits of agreement. Δ = change. Note the much narrower limits of agreement for the semi-automated (PFTquant) method

**Fig. 3:**
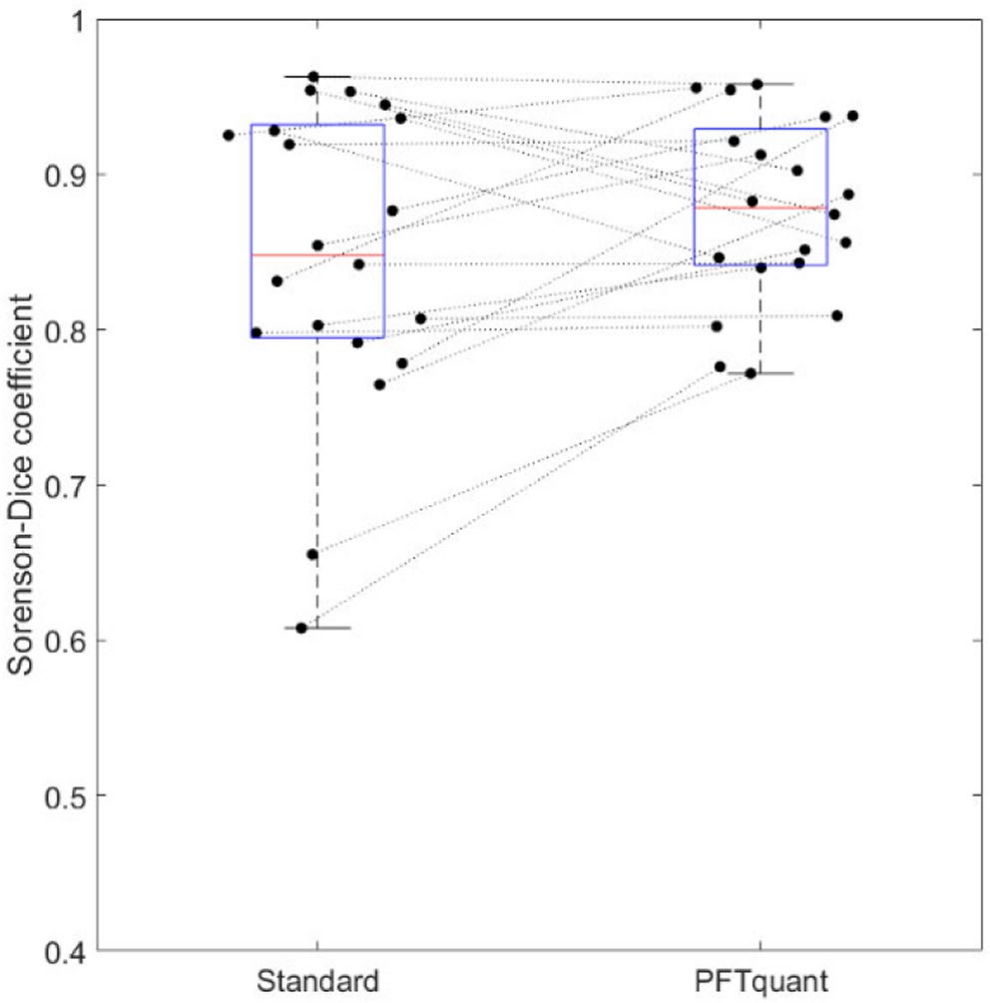
Distributions of Dice similarity coefficient (DSC) for the manual (standard) and semi-automated (PFTquant) methods of quantifying bowel fluid secretion. Each dot corresponds to the DSC between two raters for a single examination processed using the given method; lines connect the same examinations across methods

Table 1 displays summary statistics for the quantification of bowel fluid by each rater. Agreement statistics are detailed in Table 2, including agreement between manual and semi-automated image analysis by a single observer. Interrater reliability, expressed as intraclass correlation coefficients, of measured secretory response based on standard processing was moderate at 0.69 (0.36–0.86 95% C.I.) compared to excellent at 0.90 (0.76–0.96 95% C.I.) using the PFTquant software. Bland-Altman difference analysis demonstrated mean differences in measured secreted fluid volume of -0.9 mL (95% limits of agreement: -77.3–75.5 mL) and − 4.5 mL (95% limits of agreement: -39.4–30.4 mL) for standard and PFTquant processing respectively (Fig. 2). DSC were not significantly different (*p* < 0.11) for standard processing (0.85 +/- 0.10) compared to PFTquant processed cases (0.88 +/- 0.06)(Fig. 3).

**Table 1:** Bowel fluid volumes pre- and post-secretin and calculated secretory response (ΔVolume) derived from manual (standard) and semi-automated (PFTquant) MR image analysis. Results are expressed as mean +/- standard deviation (in mL) with ranges shown in parentheses

Rater	Pre-secretin volume (mL)	Post-secretin volume (mL)	ΔVolume (mL)
R1_standard_	85 ± 61 (9–203)	130 ± 74 (34–286)	46 ± 45 (-59–144)
R2_standard_	97 ± 73 (12–249)	144 ± 84 (41–340)	47 ± 52 (-82–150)
R1_PFTquant_	114 ± 86 (12–361)	169 ± 80 (42–356)	55 ± 42 (-15–136)
R2_PFTquant_	120 ± 86 (10–305)	179 ± 89 (49–357)	59 ± 36 (2–117)

R1 = rater 1 (ATT), R2 = rater 2 (JAD), standard = manual image analysis, PFTquant = semi-automated image analysis

**Table 2:** Interrater reliability statistics for pre-secretin fluid volume, post-secretin fluid volume, and change in secreted fluid volume (ΔVolume). Results are expressed as intraclass correlation coefficients with 95% confidence intervals in parentheses

Rater comparison	Pre-secretin volume	Post-secretin volume	ΔVolume
R1_standard_ vs. R2_standard_	0.83 (0.62–0.93)	0.84 (0.64–0.93)	0.69 (0.36–0.86)
R1_PFTquant_ vs. R2_PFTquant_	0.95 (0.88–0.98)	0.95 (0.87–0.98)	0.90 (0.76–0.96)
R1_standard_ vs. R1_PFTquant_	0.77 (0.40–0.91)	0.79 (0.16–0.94)	0.70 (0.39–0.87)
R2_standard_ vs. R2_PFTquant_	0.91 (0.57–0.97)	0.78 (0.37–0.92)	0.66 (0.32–0.85)

R1 = rater 1 (ATT), R2 = rater 2 (JAD), standard = manual image analysis, PFTquant = semi-automated image analysis

**Fig. 4:**
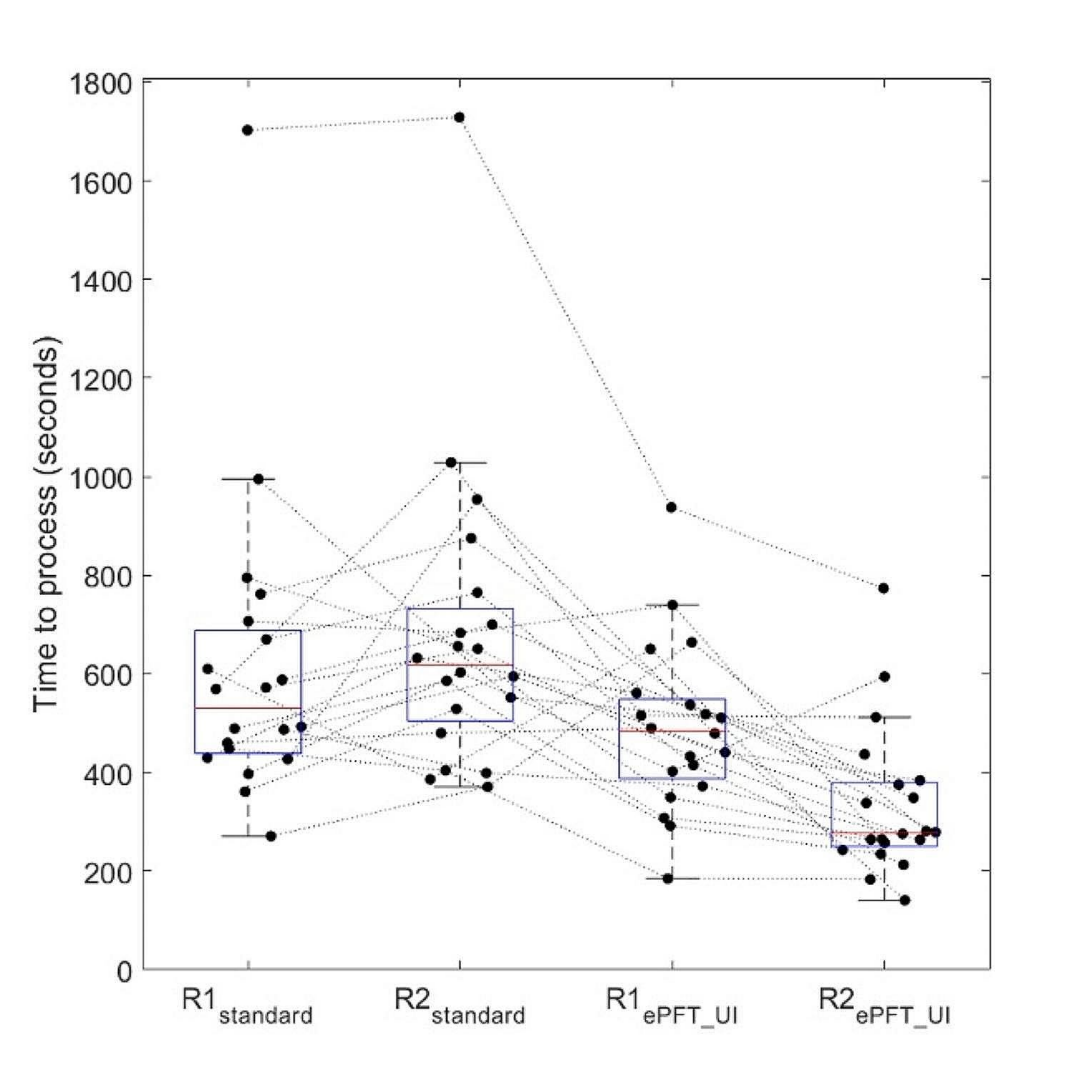
Time to process examinations was significantly shorter using the semi-automated (PFTquant) software compared to the manual (standard) technique. Each dot represents the time taken to process each exam for the given rater and method; lines connect the same exams across raters and methods. Superimposed box-whisker plots show the median (red), interquartile range (blue), and +/-2.7 times the standard deviation of time to process for each rater and method. The set of outlying dots, fully above the whiskers, reflect processing times for a patient with abundant ascites. Separating fluid in bowel from ascites required extra time with both methods but was shorter using the PFTquant

The mean time to process examinations using the standard technique (645 +/- 305 s) was significantly longer (*p* < 0.001) when compared to the PFTquant technique (412 +/- 177 s)(Fig. 4). This reflects an average time savings of 234 s, or approximately 36% time savings.

## Discussion

MR pancreatic function testing (MR-PFTs) allows non-invasive assessment of pancreatic exocrine function by subjectively characterizing or objectively quantifying fluid secreted in response to administration of a secretagogue. Quantitation of secretory response eliminates subjectivity and is particularly important in children where secreted fluid volume is known to increase with age, necessitating application of age-specific diagnostic thresholds [8]. Further, quantitation presents an opportunity to monitor changes in secretory response over time. Among the barriers to adoption of quantitative MR-PFTs is the need for time-intensive manual image segmentation and the potential for interrater variability that may impact diagnostic performance. To address the limitations and to facilitate adoption of quantitative MR-PFTs, we developed a software solution (PFTquant) designed to minimize interrater variability and reduce processing time by (1) automating initial thresholding of the MR images, (2) automatically detecting and removing hyperintense voxels that are not bowel fluid, and (3) providing semi-automated interactive tools for segmentation refinement that encourage consistent, data-driven contours. Application of PFTquant yielded substantial and significant improvements in both interrater reliability (as measured by intraclass correlation coefficients) and efficiency (as measured by time to process examinations). Importantly, these improvements were observed in a clinical population, demonstrating the relevance of this work to pancreatic disease and should encourage the wider adoption of MR-PFTs in clinical care and research. The simplicity of the workflow should also reduce barriers to adoption: a 10-minute tutorial video covers all functionality and served as the entirety of training in this work. This low learning curve should allow for the quantitation to be performed by clinical image analysts in so-called “3D labs” instead of board-certified radiologists. The PFTquant software is freely available for non-commercial use under the Creative Commons Attribution-NonCommerical license version 4.0 (CC BY-NC 4.0) and can be downloaded from the author’s GitHub (https://github.com/duddb3/PFTquant).

In our study of a sample of 20 clinically-obtained MRI examinations with MR-PFTs, we showed application of PFTquant could achieve excellent interrater agreement with a negligible mean difference (-4.5 mL) in quantified secreted fluid volume. This compared to moderate interrater agreement for manual processing of the same data sets. Further, use of PFTquant reduced the time for analysis of patient data sets by 36%. Agreement between observers in the current study for manual segmentation was less than previously demonstrated in a sample of 31 pediatric patients [10]. In that study which used a similar manual segmentation process (ImageJ), Trout et al. showed observers could achieve strong correlation (*r* = 0.92) with negligible mean bias (2 mL) in measured secreted fluid volume. Notably, the observers in that prior study were highly experienced with the technique with results likely reflecting the best-case scenario. Further, the time required to perform the manual segmentations in that prior study was not reported. To our knowledge there are no other studies with which to compare the current work.

Our study is limited by the fact that it is a single center study using a small number of MRI examinations, performed on a single vendor MRI platform, with analysis by a small number of users/observers. Use of the PFTquant requires matched image series pre- and post-secretin, acquired to optimize conspicuity of fluid content of bowel. Performance of the software using other image sets is unknown. The small number of examinations and small number of observers included may inadequately characterize the performance of the software. However, our results show meaningful improvements in efficiency and interrater reliability with application of the software in this small study.

In conclusion, we have developed a software solution to facilitate quantitative analysis of MR-PFTs which reduces the time required to process these examinations and improves interrater agreement. These are important steps to the wider adoption of MR-PFTs in clinical care and research, allowing exploration of the potential benefit or impact of quantitative MR-PFTs over qualitative secretory response assessment. Necessary future directions include application of PFTquant to MR-PFT performed in a typical/healthy pediatric population to construct normative curves of secretory response across ranges of ages and body surface area. Resultant curves can then be integrated into future versions of the software such that individual cases can be plotted against them to aid in clinical decision making.
